# Factors affecting the stability of maxillary extraction site closure

**DOI:** 10.1590/2177-6709.26.2.e2119187.oar

**Published:** 2021-05-17

**Authors:** Larissa BRESSANE, Guilherme JANSON, Rodrigo NAVEDA, Marcos Roberto de FREITAS, Daniela GARIB

**Affiliations:** 1 Universidade de São Paulo, Departamento de Ortodontia da Faculdade de Odontologia de Bauru (Bauru/SP, Brazil).; 2 Hospital de Reabilitação de Anomalias Craniofaciais (Bauru/SP, Brazil).

**Keywords:** Angle Class I malocclusion, Tooth extraction, Orthodontic space closure, Space reopening

## Abstract

**Introduction:**

A side effect observed in cases treated with extractions is the instability of orthodontic space closure.

**Objective:**

The aim of this study was to investigate the influence of gingival invagination, presence of third molars and facial pattern, on the stability of orthodontic space-closure in the maxillary arch.

**Methods:**

Ninety-nine subjects (41 male and 58 female) with Class I malocclusion treated with four premolars extraction were evaluated. Extraction sites reopening and gingival invaginations were evaluated in scanned dental models in the posttreatment and 1-year posttreatment stages (mean age 16.1 years). Third molars presence was evaluated at 1-year posttreatment panoramic radiographs, and the facial pattern (SN.GoGn) was evaluated in the initial lateral headfilms. Multiple logistic regression analysis was used to estimate the influence of the aforementioned independent variables on the frequency of extraction space reopening.

**Results:**

Space reopening was observed in 20.20% of the subjects 1-year post-debonding. Gingival invaginations were present in 25.73% of quadrants after debonding and in 22.80% 1-year posttreatment. The mean pre-treatment SN.GoGn was 35.64 degrees (SD=5.26). No significant influence was observed of the three independent variables on the instability of extraction site closure.

**Conclusions:**

The presence of gingival invaginations, third molars and facial growth pattern do not seem to influence maxillary extraction sites reopening.

## RESUMO

**Introdução:**

Um efeito colateral observado nos casos tratados com extrações é a instabilidade do fechamento ortodôntico do espaço.

**Objetivo:**

O objetivo do presente estudo foi investigar a influência da invaginação gengival, da presença de terceiros molares e do padrão facial na estabilidade do fechamento ortodôntico dos locais de extração na arcada superior.

**Métodos:**

Noventa e nove indivíduos (41 homens e 58 mulheres) com má oclusão de Classe I tratados com extração de quatro pré-molares foram avaliados. A reabertura dos locais de extração e as invaginações gengivais foram avaliadas nos modelos dentários digitalizados nos estágios pós-tratamento e um ano pós-tratamento (idade média de 16,1 anos). A presença dos terceiros molares foi avaliada em radiografias panorâmicas de um ano pós-tratamento, e o padrão facial (SN.GoGn) foi avaliado nas radiografias laterais iniciais. Análise de regressão logística múltipla foi utilizada para estimar a influência das variáveis independentes citadas na frequência de reabertura do espaço de extração.

**Resultados:**

A reabertura do espaço foi observada em 20,20% dos sujeitos um ano após a remoção do aparelho. Invaginações gengivais estiveram presentes em 25,73% dos quadrantes após a remoção do aparelho e em 22,80% após um ano pós-tratamento. O SN.GoGn pré-tratamento médio foi de 35,64 graus (DP = 5,26). Não foi observada influência significativa das três variáveis independentes sobre a instabilidade do fechamento do local de extração.

**Conclusões:**

A presença de invaginações gengivais, terceiros molares e padrão de crescimento facial não parece influenciar na reabertura dos locais de extração maxilar.

## INTRODUCTION

Maintaining extraction spaces fully closed in the long-term remains a challenge for clinical Orthodontics.^1^ ^-^ ^4^ Extraction space reopening determine both esthetic and functional problems, such as interproximal food impaction.^1^ Approximately 30% of Class I patients presented extraction space reopening 1-year posttreatment.^5^ The group with space relapse presented smaller initial dental crowding and greater amount of incisors retraction during orthodontic treatment. 

Some factors such as inadequate dental interdigitation, imbalance between intraoral and extraoral forces, deficient occlusal results after orthodontic treatment, lack of proper retention protocol, distortion of the periodontal fibers, growth pattern and root parallelism have been considered to influence the stability of closed-spaces.^1^ ^,^ ^3^ Nevertheless, reevaluation of closed-spaces stability has shown no correlation with some of these factors.^3^ ^,^ ^4^ No previous study has evaluated the influence of gingival invagination, presence of third molar and facial growth on opening of extraction space using regression analysis.

After closure of an extraction site, excess of gingival tissue appears in a papillary form between the approximated teeth.^1^ This gingival deformation, denominated gingival invagination, is not rapidly reorganized by the oral physiologic process and appears to be associated with orthodontic space relapse in extraction areas.^1^ However, the association between extraction space reopening and gingival invaginations has not been demonstrated so far.^1^ ^,^ ^3^

It has been suggested that the presence of third molars may influence the long term stability of mandibular alignment.^6^ Although there is no scientific evidence of the third molars role in orthodontic retention,^7^ some studies sustain that third molars may move teeth mesially in the long term.^8^ Considering that there is physiologic mesial movement during third molars development, these mesial forces may possibly influence the long-term stability of extraction-site closure, maintaining the spaces closed.

One essential factor for orthodontic diagnosis and prognosis is the facial growth pattern. Several studies have demonstrated greater instability of anterior dental alignment in hyperdivergent patients.^9^ ^-^ ^11^ As a dental compensation of the growth pattern, the incisors tend to develop more vertically, increasing their retroclination.^9^ Considering this long-term behavior of the anterior teeth, would hiperdivergent patients present greater stability of extraction space closure? To date there are no investigations on this matter.

Considering the elevated prevalence of extraction space reopening in the first-year posttreatment,^5^ the present study aims to assess whether gingival invagination, presence of maxillary third molars and facial growth pattern are associated with extraction space relapse in the maxillary arch.

## MATERIAL AND METHODS

This study was approved by the Ethics in Research Committee of *Faculdade de Odontologia de Bauru, Universidade de São Paulo* (protocol 45794214.1.0000.5417). The initial sample comprised orthodontic records of over 2,000 patients treated with extractions between 1973 to 2015, that were retrospectively selected from the files of *Departamento de Ortodontia da Faculdade de Odontologia de Bauru, Universidade de São Paulo*. Considering a prevalence of extraction space reopening of 30.23% (0.302)^*5*^ as *p*, and three independent variables as k, sample calculation used the formula 10 k/p, by Peduzzi et al.^*12*^ The minimum number of cases to include was 99 subjects based on an alpha significance level of 0.05 and a beta of 0.2.

The inclusion criteria were: Class I malocclusion treated with four premolars extractions; first premolars extracted in the maxillary arch; permanent dentition; maximum of 21 years of age at treatment onset; absence of number dental anomalies; no history of periodontal surgery in the extraction areas; and complete and good quality dental records, including 1-year posttreatment dental models. The first 99 patients that met the inclusion criteria were selected.

The information regarding initial, final and 1-year posttreatment ages, as well as treatment time is described in Table 1.

**Table 1:** Age distribution and treatment time

	Mean (years)	SD
Initial age	13.02	2.44
Age at the end of treatment	15.14	2.58
Age 1-year posttreatment	16.10	2.58
Treatment time	2.11	0.59

**Figure 1:**
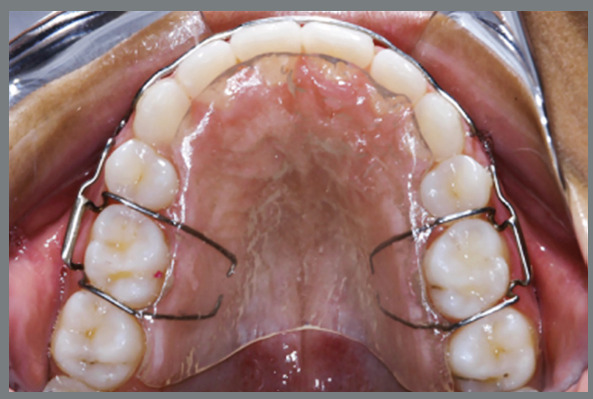
The modified Hawley retainer used by all patients in the sample.

All patients were treated by graduate students with 0.022x0.028-in fixed Edgewise appliances. Patients with severe anterior crowding required initial canine retraction. The archwire sequence for leveling and alignment was 0.015-in twist-flex or 0.016-in NiTi archwires, followed by 0.016, 0.018, 0.020, and 0.019x0.025-in stainless steel archwires. The extraction spaces were closed with *en-masse* retraction of the anterior teeth, with elastic chains on a rectangular stainless steel archwire. After the end of treatment, a modified Hawley retainer was used in the maxillary arch, and a fixed canine-to-canine archwire was bonded in the mandibular arch, as retention (Fig 1). The Hawley retainer was recommended to be used full-time for six months, followed by nights-only use for additional six months. The mandibular canine-to-canine bonded fixed retainer was recommended to be used for 3 years.

**Figure 2:**
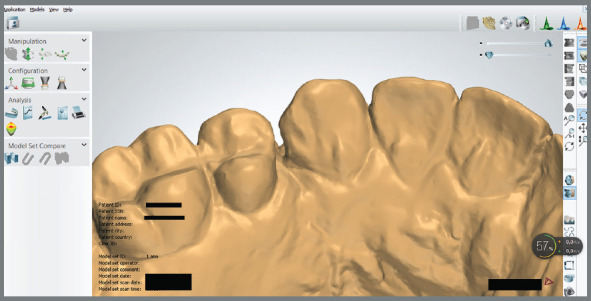
Visual evaluation of extraction space reopening.

**Figure 3:**
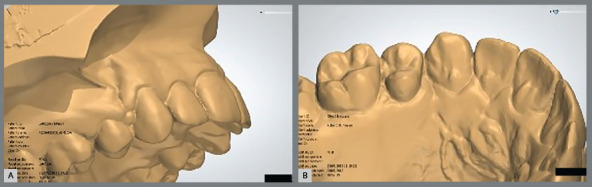
Visual evaluation of the presence of gingival invaginations in the extraction sites in the vestibular **(**A) and in the palatal **(**B) view.

Posttreatment and 1-year follow-up dental models were digitized using a 3D 3Shape R700 scanner (3Shape A/S, Copenhagen, Denmark). The following variables were evaluated using OrthoAnalyzer^TM^ 3D software (3Shape A/S, Copenhagen, Denmark):

» Extraction space reopening: the presence/absence of interproximal contact between canines and second premolars was visually performed. Patients presented extraction space reopening when a fully closed site at the end of orthodontic treatment had lost interproximal contact at the 1-year follow-up (Fig 2), independently of the amount.
» Gingival invagination: presence of gingival invagination was considered when a clear gingival fold was present in the extraction areas on the buccal and/or lingual alveolar surface (Fig 3).

The presence of maxillary third molars was assessed in the one year-posttreatment panoramic radiographs.

The initial cephalometric radiographs were digitized using the Microtek ScanMaker i800 scanner (Santa Fe Springs, CA, USA) and analyzed with Dolphin^®^ 11.5 imaging software. The magnification factors were corrected. Facial growth pattern was evaluated by the SN.GoGn angle. A trained examiner evaluated all the variables.

### ERROR STUDY

The quantitative variable SN.GoGn angle was re-measured in thirty randomly selected patients after a 30-day interval. Intraobserver random and systematic errors were calculated with Dahlberg’s formula^13^ and dependent *t*-test, respectively, at a significance level of 5%. This variable was also tested for normality using Shapiro-Wilk test.

### STATISTICAL ANALYSIS

A multiple logistic regression analysis was used to estimate the influence of each factor - gingival invagination, presence of third molars and facial growth pattern - on the occurrence of extraction space reopening. The significance level was 5%. All statistical analyses were performed with Statistica software (Statistica for Windows, version 11.0, Statsoft, Tulsa, Okla).

## RESULTS

The random error for the variable SN.GoGn was within acceptable limits (2.74)^14^ and there was no statistically significant systematic error. The SN.GoGn angle presented normal distribution.

From the 198 maxillary spaces evaluated at the end of treatment, 171 presented fully closed spaces, and were thus considered for the regression analysis. When the variables were evaluated per quadrant, only the quadrants with fully closed spaces were taken into account. Results showed that 20 out of 99 (20.20%) of the patients demonstrated that at least one extraction site reopened after 1-year follow-up (Table 2). Extraction space reopening occurred unilaterally in 15.15% of the patients and bilaterally in 5.05%. Considering the number of quadrants in the maxillary arch that were closed at the end of treatment, 25 out of 171 (14.61%) of the closed sites reopened 1-year posttreatment.

**Table 2:** Prevalence of extraction space reopening at the 1-year-posttreatment stage.

Independent variable	Sample (n=99)	Per quadrant (n=171)
Space reopening	21 (21.21%)	25 (14.61%)

The prevalence of gingival invaginations at the end of treatment in the sample subjects was 34.34% (34 out of 99 - Table 3). Considering the number of quadrants with fully-closed extraction sites, 44 out of 171 (25.73%) presented a visible gingival fold on the buccal and/or lingual alveolar surface. From the 44 sites with gingival invagination, 9 had the gingival fold both in the buccal and lingual aspects of the alveolar ridge, while 35 had the invagination only on the buccal or lingual aspects. In the 1-year posttreament dental casts, 39 out of 44 (88.63%) of the gingival invaginations persisted in the extraction sites fully closed at the end of treatment, while 11.37% disappeared.

**Table 3:** Prevalence of gingival invaginations at the end of treatment and at the 1-year-postreatment stage.

Variable	End of treatment		1-year-posttreatment
	Sample (n=99)	Per quadrant (n=171)	Per quadrant (n=44)
Gingival invaginations	34 (34.34%)	44 (25.73%)	39 (88.63%)

In the 1-year posttreatment panoramic radiographs, 90.90% of the patients had maxillary third molars present. From the 171 evaluated sites, in 154 (90.05%) the third molars were present. 

In relation to the growth pattern, the mean value for the SN.GoGn angle was 35.64° (Table 4).

**Table 4:** Distribution of SN.GoGn angle.

Variable	Mean	SD	Minimum	Maximum
SN.GoGn	35.64^0^	5.26	20.60^0^	50.50^0^

Results of the multiple logistic regression analysis showed no influence of the independent variables (gingival invagination, presence of third molars and growth pattern) on the frequency of extraction space reopening (Table 5).

**Table 5:** Multiple logistic regression analysis using the frequency of extraction space reopening as the dependent variable, and gingival invagination, presence of third molars and facial pattern as independent variables (n=171).

Independent variables	B	Standard error B	p
Gingival invagination	0.8	0.44	0.073
Presence of third molars	1.29	1.05	0.219
Facial pattern	-0.05	0.04	0.227

## DISCUSSION

From the 99 subjects selected for this study, 20.20% had at least one site with space reopening in the first-year posttreatment (Table 2). Considering the number of closed maxillary quadrants at the end of treatment (171), 14.61% presented space reopening. Previous studies have also reported the high frequency of extraction space reopening,^1^ ^,^ ^5^ encouraging the investigation of associated factors. Garib et al^5^ recently observed a 30.23-percent prevalence of extraction space reopening one year after fixed appliance removal. Given this high incidence, the first and second years of retention after appliance removal would be essential for space closure stability.^5^ ^,^ ^15^ According to Thilander et al,^16^ the orthodontist must distinguish the rapid relapse, occurring during the period of remodeling of periodontal structures, from the slow relapse, which responds to late changes occurring during the postretention period. Thus, this study evaluated the “rapid relapse” of extraction-sites reopening.

Gingival invaginations were observed in 34.34% of the subjects and in 25.73% of the quadrants, at the end of treatment (Table 3). Similarly, Robertson et al,^17^ whose investigation also included clinical observation and probing of extraction sites, found a 35-percent prevalence of gingival invaginations. On the other hand, Rivera Circuns and Tulloch^3^ found a higher prevalence of gingival invaginations (87.5%) compared to our results. These differences may rely on the methodological differences, once the gingival papilae were probed in the study by Rivera Circuns and Tulloch,^3^ and due to inclusion of the mandibular arch in the survey, where gingival invaginations are more common and severe.^3^ ^,^ ^17^ ^,^ ^18^ Late closure of extraction sites may also influence the formation and severity of gingival folds.^18^ ^,^ ^19^ Diedrich and Wehrbein^19^ compared the frequency of gingival invaginations in recent and healed extraction sites, and their results showed that early closure of the spaces reduces the occurrence of invaginations. Thus, less deleterious effects are likely to occur on attached gingiva when orthodontic retraction is performed into fresh extraction sites. However, a more recent study showed no statistically significant differences between early and late space closure regarding the incidence and severity of gingival invaginations.^20^

There was a clear tendency for gingival invaginations to persist in the long-term posttreatment. From the 44 quadrants presenting invaginations, 88.63% remained in the one-year follow-up, while 11.37% disappeared (Table 3). These findings are in accordance with the results by Robertson et al,^17^ showing that gingival invaginations may persist for as long as five years after extraction space closure. Edwards stated that, eventually, natural oral processes might completely eliminate the excess of gingival tissue between approximated teeth.^1^ The etiology of gingival invaginations seems to be related to fibers displacement instead of remodeling, and may persist for long periods after orthodontic retention.^21^

Numerous authors suggested that gingival invaginations were the main predisposing factor for extraction space reopening.^1^ ^,^ ^2^ ^,^ ^17^ ^-^ ^19^ ^,^ ^21^ ^-^ ^23^ Our results, in accordance with the study by Rivera Circuns and Tulloch,^3^ have not confirmed such assumption. No significant correlation was observed between gingival invagination and space reopening (Table 5). Thus, periodontal surgery for solving the invaginations in order to avoid space relapse is not substantiated.

The presence or absence of third molars were not related to space relapse/stability (Table 5). The possible explanation is that the irruption of third molars do not have enough force to produce mesial posterior teeth movement.^24^ ^,^ ^25^

The mean value for the SN.GoGn angle was 35.64°, indicating that the sample had a slight vertical growth tendency (Table 4).^26^ The inclusion criteria may explain this result, once patients treated with four-premolar extractions were selected. Premolar extractions are most often required in hyperdivergent patients, considering their reduced overbite, compared to hipodivergent patients.^27^ ^-^ ^30^ Hyperdivergent patients tend to end orthodontic treatment with the mandibular incisors more vertically positioned, while hypodivergent patients have more projected mandibular incisors at the end of treatment.^29^ The hypothesis that hyperdivergent patients would present greater stability of extraction space closure was rejected. No significant correlation between SN.GoGn angle, which is usually used to determine growth pattern, and extraction space reopening was found (Table 5).

Stability of extraction space closure remains uncertain, considering that most investigations that searched for associated factors did not detect significant results.^3^ However, a greater amount of initial crowding and smaller anterior retraction seem to positively influence the stability of extraction space closure.^5^ Therefore, treatment of biprotrusion performed with extractions, needing accentuated retractions, would demand longer retention time.

Despite the limitations of having evaluated gingival invaginations on dental casts, the results of this study should be considered when closed-spaces reopening is evaluated. Future studies should investigate closed-space reopening and the predisposing factors in Class II and Class III compensatory treatment.

## CONCLUSIONS

» Maxillary extraction space reopening was observed in 21.21% of the subjects and in 14.61% of the quadrants, 1-year posttreatment.
» One third of the patients had gingival invaginations on the maxillary extraction sites at the end of treatment.
» One-year posttreatment, 88.63% of the gingival invaginations persisted.
» Gingival invaginations, presence of maxillary third molars and facial growth pattern do not seem to influence space closure stability in the maxillary arch.
